# Waist circumference prediction for epidemiological research using gradient boosted trees

**DOI:** 10.1186/s12874-021-01242-9

**Published:** 2021-03-09

**Authors:** Weihong Zhou, Spencer Eckler, Andrew Barszczyk, Alex Waese-Perlman, Yingjie Wang, Xiaoping Gu, Zhong-Ping Feng, Yuzhu Peng, Kang Lee

**Affiliations:** 1 Department of Health Management Centre, Drum Tower Hospital Affiliated to Nanjing University Medical School, No. 321 Zhongshan Road, Nanjing, 210008 China; 2 Dr. Eric Jackman Institute of Child Study, University of Toronto, 45 Walmer Rd, Toronto, ON M5R 2X2 Canada; 3 Department of Physiology, University of Toronto, Medical Sciences Building, Rm. 3306. 1 King’s College, Toronto, ON M5S 1A8 Canada; 4 Department of Anaesthesiology, Drum Tower Hospital Affiliated to Nanjing University Medical School, No. 321 Zhongshan Road, Nanjing, 210008 China

**Keywords:** Waist circumference, Machine learning, Gradient boosted trees, Multilayer perceptron

## Abstract

**Background:**

Waist circumference is becoming recognized as a useful predictor of health risks in clinical research. However, clinical datasets tend to lack this measurement and self-reported values tend to be inaccurate. Predicting waist circumference from standard physical features could be a viable method for generating this information when it is missing or mitigating the impact of inaccurate self-reports. This study determined the degree to which the XGBoost advanced machine learning algorithm could build models that predict waist circumference from height, weight, calculated Body Mass Index, age, race/ethnicity and sex, whether they perform better than current models based on linear regression, and the relative importance of each feature in this prediction.

**Methods:**

We trained tree-based models (via XGBoost gradient boosting) and linear models (via regression) to predict waist circumference from height, weight, Body Mass Index, age, race/ethnicity and sex (*n* = 60,740 participants). We created 10 iterations of each model, each using 90% of the dataset for training and the remaining 10% for testing performance (this group was different for each iteration). We calculated model performance and feature importance as an average across 10 iterations. We then externally validated the ensembled version of the top model.

**Results:**

The XGBoost model predicted waist circumference with a mean bias ± standard deviation of 0.0 ± 0.04 cm and a root mean squared error of 4.7 ± 0.05 cm, with performance varying slightly by sex and race/ethnicity. The XGBoost model showed varying degrees of improvement over linear regression models. The top 3 predictors were Body Mass Index, weight and race (Asian). External validation found that on average this model overestimated waist circumference by 4.65 cm in the United Kingdom population (mainly due to overprediction in females) and underestimated waist circumference by 1.7 cm in the Chinese population. The respective root mean squared errors were 7.7 cm and 7.1 cm.

**Conclusions:**

XGBoost-based models accurately predict waist circumference from standard physical features. Waist circumference prediction using this approach would be valuable for epidemiological research and beyond.

**Supplementary Information:**

The online version contains supplementary material available at 10.1186/s12874-021-01242-9.

## Background

Epidemiological research studies often assess adiposity and its associated health risks from a standard set of physical features. Body Mass Index (BMI) is a measure of general adiposity and is calculated from height and weight and assessed against age and sex-based disease risk thresholds. It predicts generalized mortality [1] and illnesses like colon cancer [2] and incident diabetes [3]. New indices like Body Shape Index and Waist-to-Height ratio have respectively improved predictions of mortality [4] and cardiometabolic risk [5] in recent years. Both indices require waist circumference. However, since the value of waist circumference has become apparent only recently, it has not been routinely collected in clinical practice [6] and it is less likely to appear in clinical databases. Even when waist circumference information is present, it is frequently collected through self-report, which despite being highly accurate for weight and height is notoriously inaccurate for waist circumference [7]. This limits the use of these new and improved indices.

Existing methods of dealing with missing waist circumference values have limitations. Excluding subjects without waist circumference information limits sample size and in many cases affects sample representativeness; often epidemiological research depends on representative population samples. Mean replacement is undesirable because it provides no information about individual subjects. Multiple imputation is a common way of estimating missing values within a dataset based on a subject’s covariate information and uses Bayesian regression techniques. Finally, some studies have used standard linear regression to deliberately model the relation between readily available covariates and waist circumference to estimate missing waist circumference values or evaluate the accuracy of self-reported waist circumference values. Bozeman et al. [8] have formalized such an approach and derived a dedicated equation to predict waist circumference based on Body Mass Index, age, sex, and race/ethnicity. While this approach was highly accurate, its accuracy may nevertheless be limited by not accounting for the possibility of non-linear relations and interactions between variables (the Bozeman approach accounts for just one non-linearity: it derives separate coefficients for female ages above and below 35). It does not consider the non-linearity of Body Mass Index with waist circumference (opting instead to exclude subjects with Body Mass Indexes over 40), and it does not consider potential interactions among predictors (e.g., with respect to race) other than for sex, which it addresses by deriving separate equations for males and females. Such relations occur frequently among variables within biological systems, but they are not frequently investigated.

This issue could be addressed with a machine learning approach. Advanced machine learning algorithms make it possible to automatically and efficiently model complex non-linear relations and interactions involving multiple predictors [9]. This automaticity of modeling is beneficial compared to traditional statistical regression that requires explicit modeling of each relation since it increases the likelihood of identifying novel complex relations (particularly subtle ones) that are not feasible to model manually. This increases the comprehensiveness of the model and thus improves prediction accuracy. The large datasets characteristic of epidemiological studies provide the statistical power necessary to identify these novel and subtle relations in clinically diverse populations [10]. Compared to traditional statistical modeling techniques, machine learning models allow more flexibility in mapping inputs to outputs (compared with deterministic functions), and they are not subject to potentially invalid assumptions like homoscedasticity in the data [11]. Thus, they can potentially be more precise and generate more accurate predictions, while still providing interpretability through calculations of predictor importance. It has yet to be determined how predictive models for waist circumference perform using advanced machine learning techniques.

This study investigated the degree to which the combination of height, weight, Body Mass Index (calculated from height and weight), age, race/ethnicity, and sex predict waist circumference using machine learning. We trained a predictive model using an XGBoost machine learning algorithm and then validated its accuracy on an independent portion of the dataset that was not used in training. To determine whether this model adequately accounted for sex differences, we also trained and tested separate models for males and females and compared their performance to the sex-aggregated model. To determine whether our machine learning approach was beneficial compared to traditional statistical regression, we trained and tested 3 regression models (a linear regression using a semi-Bayesian ridge regression technique, a linear regression using the Bozeman technique, and a linear regression incorporating all possible predictor interactions) and compared their performance to our sex-aggregated XGBoost model. We evaluated model performance overall, by sex, and within sex-race groups. We anticipated that all models would yield highly accurate predictions (based on good past performance with linear models using similar predictors [8]), and that our sex-aggregated XGBoost model would perform similarly to sex-specific XGBoost models and better than any of the linear regression approaches. Finally, we externally validated an ensemble of our sex-aggregated XGBoost models on two external datasets to determine how they perform on disparate populations.

## Methods

### Participants

For model building and internal validation we obtained height (centimeters), weight (kilograms), age (years), race/ethnicity (mutually exclusive categories were Asian, Black, white, Hispanic (of any race), with each coded as a binary variable; other/mixed race served as the baseline), sex (male/female), and waist circumference (centimeters) information for 60,740 subjects from the physical examination records of Chinese patients at Nanjing Drum Tower Hospital (affiliated with Nanjing University Medical School) and from publicly available American data collected in the 1999–2017 survey years of the National Health and Nutrition Examination Survey (NHANES) [12]. Chinese data was collected in conjunction with routine annual physical examinations and thus these patients were considered generally healthy. All subjects met the following inclusion criteria: (1) only one record per participant was used (if there were multiple visits on file), (2) complete information for all variables, (3) an age of 18 or older, and (4) no outliers (defined as 3 standard deviations above or below the mean). Participants provided written informed consent to have their data used in the study and the study protocol was approved by the Nanjing University Research Ethics Board and the National Center for Health Statistics Research Ethics Review Board, respectively.

Study participants were 56% male and 44% female. With respect to race/ethnicity, 42% were Asian, 14% were black, 16% were Hispanic 1% were of mixed/other race and 27% were white. Means and standard deviations for age, height, weight, and waist circumference were 49.0 ± 17.6 years, 167.7 ± 9.3 cm, 75.6 ± 16.7 kg, and 92.6 ± 14.4 cm, respectively.

For external validation we obtained nationally representative data for the United Kingdom from the 2000–2001 survey year of the National Diet and Nutrition Survey (NDNS) [13], and nationally representative data for China from the 2015 survey year of the China Health and Nutrition Survey (CHNS) [14]. Participants provided written informed consent to have their data used in the study and the study protocol. NDNS received ethics approval from the Multi-centre Research Ethics Committee (MREC) and National Health Service Local Research Ethics Committees (LRECs). CHNS received ethics approval from the Institutional Review Board of the University of North Carolina at Chapel Hill, the National Institute for Nutrition and Food safety at China Center for Disease Control and Prevention, and the Human and Clinical Research Ethics Committee of the China-Japan Friendship Hospital. We recalibrated sample weights to ensure representativeness by sex-race/ethnicity group after removing samples with missing data (78% of data remained in NDNS; 85% in CHNS). Waist circumference in these datasets was reliably measured by a trained professional.

### Waist circumference prediction models

#### Model types

We trained and tested several types of models to predict waist circumference from a set of predictors that included: height, weight, Body Mass Index (calculated from weight and height as kg/m^2^), age, race/ethnicity, and sex. For the machine learning model, we initially considered neural network-based models via the multilayer perceptron algorithm and decision tree-based models via Random Forest and XGBoost algorithms. All are well established supervised learning algorithms that allow modeling of non-linear relations and interactions among predictors. We ultimately selected gradient-boosted trees via the XGBoost algorithm because empirically this performed best in our preliminary investigation. We ultimately used XGBoost to train a sex-aggregated model using all predictors, as well as separate models for each sex separately. We did this so we could compare their performance and confirm that the aggregated model adequately accounted for sex differences.

To assess the potential advantages of machine learning over traditional approaches, we further trained and evaluated the performance of three regression models: semi-Bayesian ridge regression (commonly employed in multiple imputation of missing values), linear regression using the current gold standard Bozeman technique, and linear regression incorporating all potential interactions among predictors so that we could determine whether XGBoost model performance was superior. All three were sex-specific models so they could account for known sex differences. We used the semi-Bayesian ridge regression method (BayesianRidge function, scikit-learn 0.24.1 package; https://scikit-learn.org/) to generate a distribution of predictions and then took the mean of that prediction (it was semi-Bayesian in that an expected distribution was not supplied a priori). The Bozeman technique was a linear regression that did not include height and weight, but included separate age terms (coefficients) for females under 35 years of age and females 35 years of age or older. Our linear regression considered all predictors and all interactions among predictors to maximize information gain from predictor interactions. Within each sex, these interactions included 2-way up to 5-way predictor-predictor interaction terms. We selected terms with coefficient *p*-values < .05 for inclusion in the final model. Such terms had robust (statistically significant) correlations with waist circumference that are likely to generalize well beyond any one sample to positively impact model performance.

#### Training and testing paradigm

We randomly allocated participants into ten equally sized folds. We then used a 10-fold cross-validation technique to train and test 10 iterations of each model. For each of the 10 iterations, 9 of 10 folds were used to train the model and the remaining fold was used to assess model performance. Calculating accuracy on this unused portion of the data that was not used in training ensured that estimates of model performance for each iteration were not influenced by overfitting to the training data. We then calculated performance as the mean across all 10 model iterations. This provided an estimate of overall performance that was generalizable to the entire dataset (rather than just one data partition) and ultimately to populations that are represented by the dataset.

#### XGBoost model and training parameters

XGBoost (Extreme Gradient Boosting; https://github.com/dmlc/xgboost) is a machine learning algorithm for building gradient-boosted decision trees and it is well-suited for regression [15]. Briefly, the algorithm builds an initial decision tree. It then builds subsequent decision trees to predict the residuals (errors) after applying the first tree. It scales this residual by a pre-defined learning rate (lower learning rates favor less variance/overfitting), and then adds its the scaled residual to the original prediction to produce a slightly improved prediction. It goes on to build additional trees in this way to incrementally increase the prediction accuracy until it reaches a pre-defined number of trees (determined as the number of trees at which additional trees no longer improve performance). *Extreme* gradient-boosted trees (in XGBoost) are unique in that they cluster residuals into leaves (determine branch splits) based on similarity scores. Groups of leaves are pruned if the gain in the group’s similarity versus the parent leaf does not exceed a user-defined value (lambda); this is a form of regularization intended to reduce overfitting. We empirically determined the best parameters to be a maximum tree depth of 5, 1000 trees, 80% sub-sampling and a learning rate of .02. The number of trees was selected empirically by increasing the number of trees by a factor of 10 until there was no additional gain in performance, and then reducing the number of trees until performance began to decline.

### Assessment of model performance

We assessed the performance of each model by calculating the mean and standard deviation of various performance metrics across the 10 iterations of each model. Performance metrics calculated for all models were root mean squared error (RMSE) and mean bias (prediction minus the reference value). They were calculated overall, for males, for females, and for each sex-race/ethnicity combination. We used a t-test to determine whether the performance of a given model statistically differed from the performance of our sex-aggregated XGBoost model (comparator model).

For the sex-aggregated XGBoost model we determined additional performance metrics and model details. We calculated the mean ± 95% confidence interval for mean bias, error standard deviation and Pearson correlation. Pearson correlation and its standard deviation were calculated by taking the square root of the mean explained variance and its standard deviation across all 10 model iterations. We visualized performance over the full range of waist circumferences using a scatter plot and Bland Altman plot to assess correlation and agreement, respectively. Finally, we calculated proportional importance of each model predictor using a function built into the XGBoost analytical package and reported its mean and 95% confidence interval.

### External validation

We further assessed the performance of the sex-aggregated XGBoost model on two nationally representative external datasets: the National Diet and Nutrition Survey (United Kingdom) [13] and China Health and Nutrition Survey (The People’s Republic of China) [14]. We took the mean of the predictions from each of the 10 versions of the model (each derived on a different fold of the training dataset) to generate an ensembled prediction for the model. We then calculated the RMSE and mean bias overall, by sex and by sex-race group for each dataset. These calculations considered sample weights and thus were nationally representative.

## Results

### XGBoost outperforms regression models

We assessed whether the waist circumference model trained with XGBoost would outperform linear regression models in terms of error (RMSE) or mean error (bias). We found that the XGBoost model trained on both males and females (sex-aggregated) achieved an RMSE of 4.70 ± 0.05 and a bias of 0 ± 0.04 overall (a further breakdown of performance by sex and race is shown in Table 1). The two models trained on each sex separately did not perform statistically better than the sex-aggregated model, and so we used the sex-aggregated model moving forward.

**Table 1:** Model performance comparison between sex-aggregated XGBoost, sex-separated XGBoost, semi-Bayesian ridge regression, Bozeman linear regression and linear regression with all possible interaction terms

Model:		XGBoost				Semi-Bayesian Ridge Regression		Bozeman Linear Regression		Linear Regression	
Sex:		Aggregated		Separate Models		Separate Models		Separate Models		Separate Models	
	Count	RMSE	Bias	RMSE	Bias	RMSE	Bias	RMSE	Bias	RMSE	Bias
**Overall**	60,740	4.70 ± 0.05	0 ± 0.04	4.71 ± 0.04 0%	0 ± 0.05	4.89 ± 0.05*** 4%	0 ± 0.04	5.01 ± 0.06*** 7%	0 ± 0.04	4.72 ± 0.05 0%	0 ± 0.04
**Female**	26,750	5.41 ± 0.09	0.01 ± 0.07	5.43 ± 0.09 0%	0 ± 0.08	5.67 ± 0.1*** 5%	0 ± 0.07	5.95 ± 0.12*** 10%	0 ± 0.06	5.46 ± 0.1 1%	0 ± 0.06
Asian	8402	4.4 ± 0.13	0.07 ± 0.17	4.39 ± 0.12 0%	− 0.01 ± 0.16	4.7 ± 0.12*** 7%	0.68 ± 0.16***	4.67 ± 0.12*** 6%	0 ± 0.16	4.54 ± 0.11* 3%	0 ± 0.17
Black	4321	5.94 ± 0.16	−0.06 ± 0.23	5.98 ± 0.17 1%	0.05 ± 0.2	6.24 ± 0.23** 5%	− 0.89 ± 0.23***	6.67 ± 0.23*** 12%	0.01 ± 0.29	5.91 ± 0.16 0%	0.01 ± 0.22
Hispanic	5298	5.62 ± 0.31	−0.01 ± 0.36	5.65 ± 0.32 1%	− 0.01 ± 0.37	5.83 ± 0.34 4%	− 0.41 ± 0.36*	6.12 ± 0.31** 9%	0 ± 0.38	5.65 ± 0.31 1%	0 ± 0.35
Other/Mixed	343	5.66 ± 0.68	− 0.83 ± 0.64	5.66 ± 0.56 0%	−0.55 ± 0.57	5.61 ± 0.79 − 1%	−0.91 ± 0.7	6.2 ± 1.15 10%	0.05 ± 0.67**	5.76 ± 0.66 2%	− 0.02 ± 0.8*
White	8386	5.87 ± 0.16	0.04 ± 0.23	5.9 ± 0.15 0%	0 ± 0.24	6.12 ± 0.19** 4%	0.08 ± 0.25	6.53 ± 0.17*** 11%	0 ± 0.27	5.9 ± 0.15 0%	0 ± 0.23
**Male**	33,990	4.05 ± 0.05	− 0.01 ± 0.07	4.05 ± 0.05 0%	0 ± 0.06	4.18 ± 0.05*** 3%	0 ± 0.07	4.13 ± 0.05** 2%	0 ± 0.06	4.04 ± 0.05 0%	0 ± 0.06
Asian	17,056	3.90 ± 0.08	− 0.02 ± 0.12	3.89 ± 0.08 0%	0.01 ± 0.11	3.98 ± 0.08* 2%	− 0.33 ± 0.11***	4 ± 0.07 ** 3%	0 ± 0.11	3.9 ± 0.08 0%	0 ± 0.12
Black	3951	4.40 ± 0.24	0.13 ± 0.19	4.4 ± 0.23 0%	0.05 ± 0.19	4.8 ± 0.22** 9%	0.99 ± 0.2***	4.56 ± 0.25 4%	0 ± 0.19	4.37 ± 0.24 − 1%	0 ± 0.19
Hispanic	4691	3.88 ± 0.12	− 0.02 ± 0.14	3.88 ± 0.13 0%	− 0.02 ± 0.15	3.96 ± 0.12 2%	0.47 ± 0.16***	3.9 ± 0.13 0%	0 ± 0.15	3.87 ± 0.12 0%	0 ± 0.15
Other/ Mixed	381	4.52 ± 0.58	−0.25 ± 0.7	4.6 ± 0.62 2%	− 0.33 ± 0.63	4.75 ± 0.64 5%	0.67 ± 0.73*	4.7 ± 0.64 4%	1.2 ± 0.68***	4.52 ± 0.51 0%	−0.03 ± 0.69
White	7911	4.25 ± 0.12	− 0.05 ± 0.1	4.25 ± 0.12 0%	− 0.01 ± 0.09	4.37 ± 0.13* 3%	− 0.08 ± 0.09	4.28 ± 0.12 1%	−0.06 ± 0.09	4.23 ± 0.1 0%	0 ± 0.09

Values represent mean ± standard deviation across the 10 iterations of each model

Percentage change for RMSE is relative to sex-aggregated XGBoost model

*RMSE* root mean squared error

*p < .05, **p < .01 and ***p < .001 for statistical significance by two-tailed t-test versus sex-aggregated XGBoost model

The root mean square error (RMSE) of all models was either equivalent to or higher than that of the sex-aggregated XGBoost model overall, by sex, and across all sex-race/ethnicity categories. The semi-Bayesian ridge regression and Bozeman regression models had higher RMSEs overall (4.89 ± 0.05 cm; *p* < .001 and 5.01 ± 0.06 cm; *p* < .001, respectively). RMSEs in the Bozeman regression models were as much as 12% higher than in the XGBoost model for some sex-race/ethnicity categories. Our linear regression model with all predictor interactions performed only marginally worse than our sex-aggregated XGBoost model. Its overall RMSE overall (4.72 ± 0.05 cm) was statistically equivalent. It was statistically higher for the Asian female group, although the difference was small (only 3%). The mean bias of all models was minimal (< 1 cm), except for the Bozeman regression, which overpredicted waist circumference by 1.2 cm in males of mixed/other race. Overall, the relative superiority of XGBoost in terms of error justifies its use in place of linear regression where the minimization of error is concerned.

### Model performance details

**Fig. 1:**
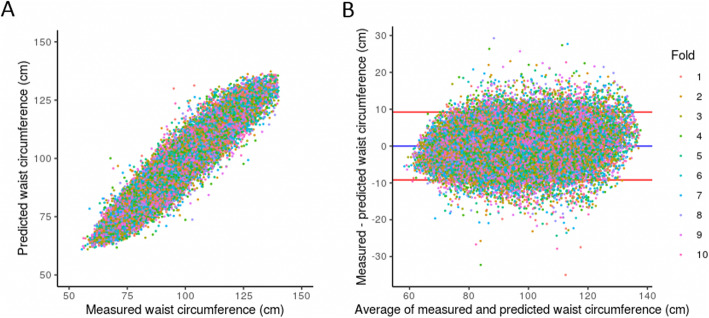
Model performance for all validation folds of the sex-aggregated XGBoost model. **a** Scatter plot of measured versus predicted waist circumference. **b** Bland-Altman plot. The blue line represents the mean bias and the red line represents one standard deviation from the mean

Overall, the sex-aggregated model performed similarly from one validation split to the next (see Fig. 1 and Supplemental Table 1). The mean Pearson correlation (95% confidence interval) between observed and predicted values was 0.945 (0.944–0.946) and the mean bias (95% confidence interval) ± error standard deviation (95% confidence interval) between observed and predicted values was − 0.003 (− 0.032–0.027) ± 4.70 (4.67–4.73) cm over all 10 iterations of the model. Predictions tracked observed values well in all validation splits across the full range of waist circumferences, as assessed visually by correlation and agreement (Fig. 1).

**Fig. 2:**
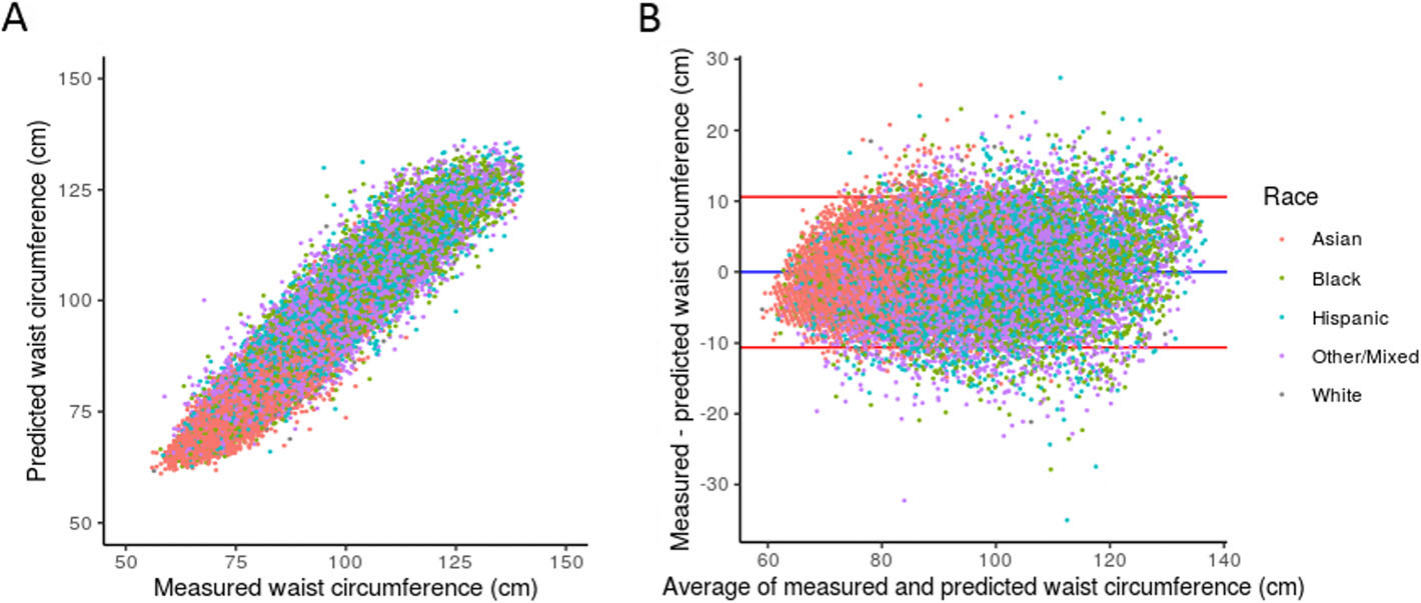
Female performance by race/ethnicity in the sex-aggregated XGBoost model. **a** Scatter plot of measured versus predicted waist circumference. **b** Bland-Altman plot. The blue line represents the mean bias and the red line represents one standard deviation from the mean

For females specifically, the mean Pearson correlation (95% confidence interval) between observed and predicted values was 0.942 (0.940–0.944) and the mean bias (95% confidence interval) ± standard deviation (95% confidence interval) between observed and predicted values was − 0.018 (− 0.058–0.095) ± 5.41 (5.35–5.48) cm over all 10 iterations of the model. Predictions tended to track observed values quite well across a range of waist circumferences, as assessed by correlation and agreement (Fig. 2). Asian females tended to have smaller waist circumferences (Fig. 2) and proportionally smaller errors (Table 1). The largest errors tended to be in black females (Table 1).

**Fig. 3:**
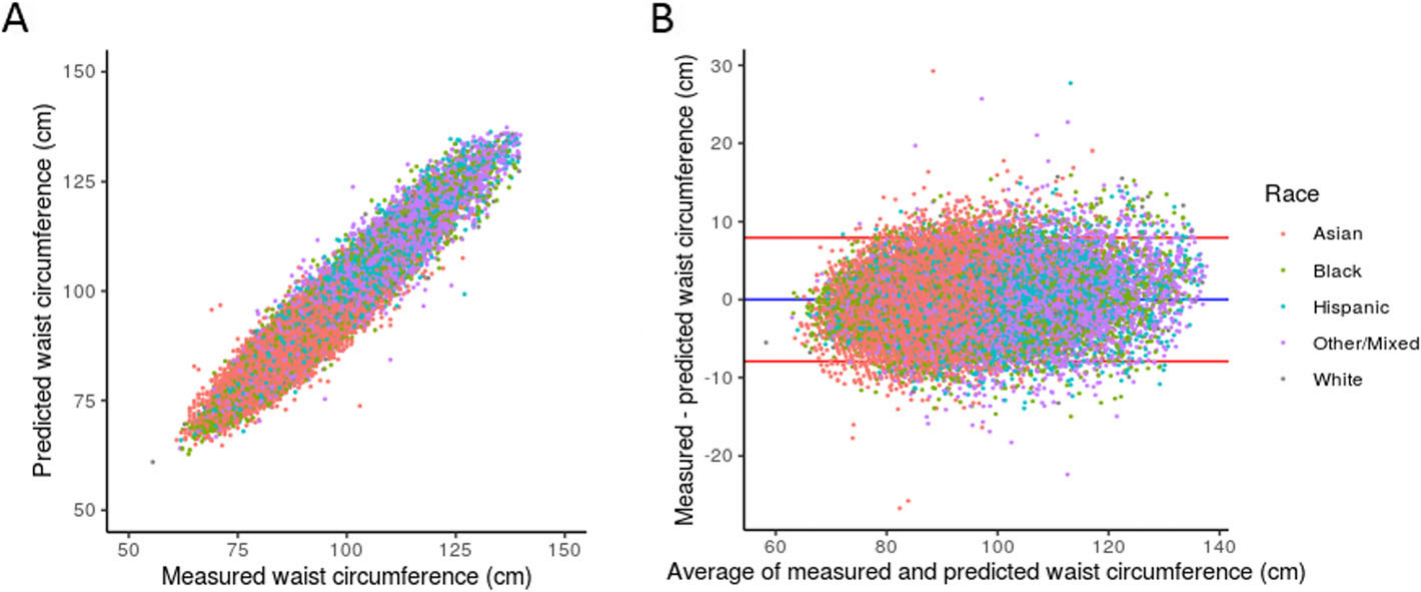
Male performance by race/ethnicity for the sex-aggregated XGBoost model. **a** Scatter plot of measured versus predicted waist circumference. **b** Bland-Altman plot. The blue line represents the mean bias and the red line represents one standard deviation from the mean

For males specifically, the mean Pearson correlation (95% confidence interval) between observed and predicted values was 0.947 (0.947–0.949) and the mean bias (95% confidence interval) ± standard deviation (95% confidence interval) between observed and predicted values was − 0.014 (− 0.061–0.034) ± 4.05 (4.01–4.08) cm over all 10 iterations of the model. Predictions tracked observed values well across the full range of waist circumferences, as assessed by correlation and agreement (Fig. 3). As with females, Asian males tended to have smaller waist circumferences (Fig. 3) and proportionally smaller errors (Table 1). Hispanic males also had proportionally smaller errors (Table 1).

### Feature importance and model details

**Fig. 4:**
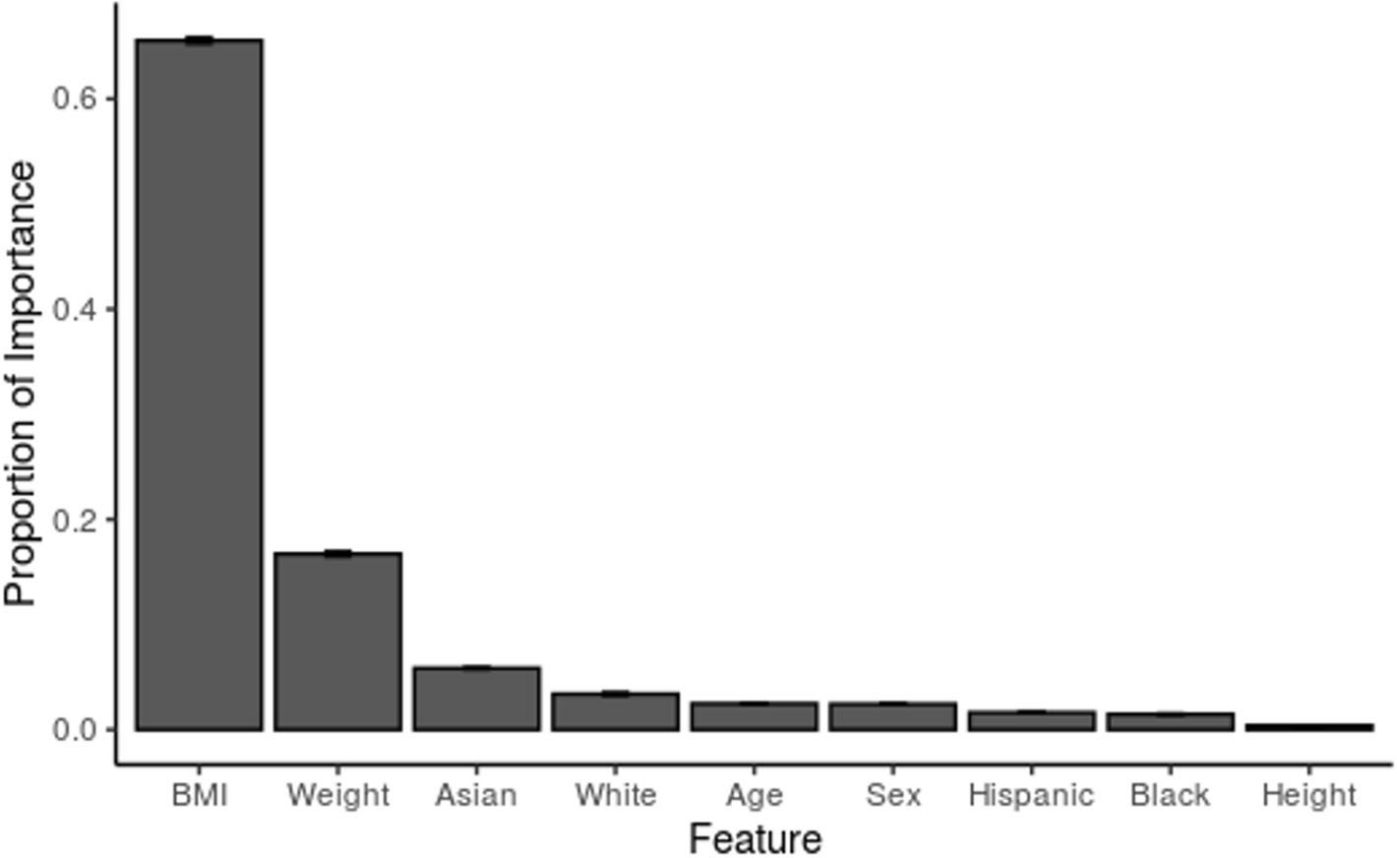
Feature importance. Feature importance is expressed as a proportion of total feature importance. Error bars are the 95% confidence interval

We next calculated the mean proportional importance (± 95% confidence interval) for all predictors in the sex-aggregated XGBoost model across all 10 iterations of the model. Body Mass Index (65.6% ± 0.3%), weight (16.8% ± 0.2%) and Asian race (5.9% ± 0.1%) were the most important features for predicting waist circumference (Fig. 4). The remaining features of white race, age, sex, Hispanic ethnicity, Black race and height all had less than 5% feature importance. For a sample decision tree diagram, see Supplementary Fig. 1.

### External validation of aggregated XGBoost model

We took the mean of the predictions from the 10 versions of the sex-aggregated XGBoost model to produce a single (ensembled) waist circumference for each prediction, and then evaluated the accuracy of this overall model in representative datasets of the United Kingdom and China (Table 2). We found that waist circumference was overestimated by 4.63 cm on average in the United Kingdom multi-racial sample; this positive bias arose mainly from a tendency to overpredict in females. There was a small negative bias of − 1.7 cm overall in the Chinese dataset. RMSEs for both external samples were comparable and approximately 50% larger than the overall RMSE calculated for the internal validation of the sex-aggregated XGBoost model.

**Table 2:** Performance of the ensembled sex-aggregated XGBoost model on external datasets

Model:	United Kingdom (National Diet and Nutrition Survey; 2000–2001)			People’s Republic of China (China Health and Nutrition Survey; 2015)		
	Proportion of Dataset	RMSE	Bias	Proportion of Dataset	RMSE	Bias
**Overall**	100	7.65	4.63	100	7.1	−1.7
**Female**	54.7	9.26	7.34	53.5	7.45	−2.02
Asian	0.4	6.4	4.92	53.5	7.45	−2.02
Black	1.2	4.81	2.85	–	–	–
Hispanic	0.9	11.18	9.07	–	–	–
Other/Mixed	1	11.39	8.18	–	–	–
White	51.3	9.31	7.47	–	–	–
**Male**	45.3	5.41	1.73	46.5	6.67	−1.34
Asian	0.5	4.13	0.34	46.5	6.67	−1.34
Black	1	6.68	−3.08	–	–	–
Hispanic	0.2	2.57	2.37	–	–	–
Other/Mixed	0.8	4.21	1	–	–	–
White	42.7	5.42	1.87	–	–	–

Values represent sample-weighted mean bias or RMSE across the entire dataset

*RMSE* root mean squared error

## Discussion

Our XGBoost model predicted waist circumference with high accuracy from height, weight, Body Mass Index, age, race/ethnicity and sex information. We found that it performed quite well across various sex-race/ethnicity groups, and very consistently across all 10 model iterations. Together, these features were highly predictive of waist circumference and the XGBoost algorithm is an effective way to model these relations. This was expected given that similar predictors already produced good performance in prior work using linear models [8]. We found that XGBoost performed significantly better than semi-Bayesian ridge regression and the Bozeman linear regression approach [8], likely because the machine learning aspect identified non-linear relations or interactions that accounted for additional variance; such relations (particularly non-linearities) are often subtle and could be difficult to deliberately identify and input as features. There was also improvement to a lesser degree against the linear regression model that considered all possible predictor interactions. Improvement in this case was likely lower because this model accounted for additional variance by considering all possible predictor interactions.

Sex-aggregated model performance for males and females did not significantly differ from sex-specific model performance, as expected. This suggests that sex-based differences in waist circumference have been adequately accounted for in the sex-aggregated model and that the sex-aggregated model can be used going forward.

Finally, we found that an ensemble of the sex-aggregated XGBoost model generalized reasonably well overall to a representative multi-racial sample of the United Kingdom; the model performed worse in females (particularly Hispanic females). The ensemble performed quite well in a representative sample of the Chinese population. The tendency to overestimate waist circumference in United Kingdom females (particularly of white, Hispanic and mixed/other backgrounds) did not occur in United States females from our original (internal) dataset and potentially suggests the presence of a sociocultural factor that could account for this difference.

### Limitations

Even greater accuracy might be achieved by further optimizing the machine learning algorithm via its hyperparameters. Additional features could also be considered to see if they add information about waist circumference and thus boost accuracy. The models in the current study are based on multiple races/ethnicities in participants with American and Chinese nationalities; further work along these lines could investigate performance in additional races/ethnicities and nationalities to determine how well this model generalizes beyond the groups studied. Nevertheless, the *technique* we have utilized in this study is likely to be effective for training generalizable predictive models using a diverse set of participants.

It further remains to be determined whether the relative performance of the methods in the current study will generalize to still other training datasets. We anticipate it would generalize well given the high degree of heterogeneity in the current training data, which encompasses significant biological diversity by being multi-racial, and significant of sociocultural diversity given that data comes from both the United States and China (two highly disparate populations). Further, the model performed fairly well when externally validated on datasets from the United Kingdom and China. Further evaluation in additional populations will be necessary to determine the accuracy of this approach elsewhere and potentially identify additional relevant factors that could affect waist circumference prediction.

### Application

The high accuracy of the sex-aggregated XGBoost model makes it suitable for various purposes. First, it can be used to fill in missing waist circumferences in medical datasets. Care should be taken with such an approach since individuals who are missing waist circumference or its predictor information may be statistically different in some way from those who are not (e.g., if their data is missing not at random). Such a hypothesis could be tested using logistic regression to try to separate subjects with missing waist circumferences from subjects without missing waist circumferences using auxiliary variables, and then recalibrating sample weights on non-missing observations accordingly to maintain sample representativeness. Second, rather than filling in missing data, the current model could be used to screen existing waist circumference measurements for errors, given that self-reported waist circumference measurements tend to be inaccurate [7]. In this case, it is beneficial to build a well-defined externally validated model using datasets with reliable waist circumference information to apply to the target dataset. If predicted waist circumference is being used as a covariate in a regression model or mathematical equation, it should be considered how the accuracy of the waist circumference prediction will carry forward to impact the accuracy of the overall regression model or equation.

Finally, we acknowledge that since the accuracy of the XGBoost model is only marginally better than the linear regression with all interaction terms, it may remain preferable in some applications to sacrifice this small degree of added accuracy in favor of the more simplistic linear regression model.

Waist circumference can ultimately be used to help predict general mortality [1] and specific illness such as cancers [2], cardiovascular diseases [5] and incident diabetes [3]. Future studies could assess the impact of using predicted waist circumference in evaluating health risks. Accurate waist circumference prediction achieved using machine learning could vastly aid future epidemiological research, particularly when attempting to predict long-term health outcomes.

## Conclusions

This study demonstrates that the XGBoost advanced machine algorithm produces accurate waist circumference prediction models with minimal input data: height, weight, Body Mass Index (calculated from height and weight), age, race/ethnicity and sex. This approach could be immensely useful in epidemiological research, in health-evaluative tools and potentially even for non-health-related uses.

## Supplementary Information

**Additional file 1: Supplemental Table 1.** Training and testing statistics for sex-aggregated XGBoost model.

**Additional file 2: Supplemental Figure 1.** Example of one decision tree (out of 1000) in the first iteration of the gender-aggregated XGBoostmodel. Nodes contain cut-offs for the variable of interest and leaves contain output values.

## Data Availability

Due to the nature of this research and privacy concerns, participants from the Nanjing Drum Tower Hospital did not agree for their data to be shared publicly, so supporting data is not available. National Health and Nutrition Survey datasets are freely available from the National Center for Health Statistics (United States of America) [12], the China Health and Nutrition Study dataset is freely available from the University of North Carolina Population Center [14], and the 2000–2001 year of the National Diet and Nutrition Survey is available from the Office for National Statistics (Social and Vital Statistics Division, Food Standards Agency), UK Data Service [13].

## Abbreviations

BMI: Body Mass Index
cm: Centimeters
RMSE: Root mean squared error
