# Supplementary material for: Waist circumference prediction for epidemiological research using gradient boosted trees
Source: BMC Med Res Methodol. 2021 Mar 9;21:47. doi: 10.1186/s12874-021-01242-9 (PMC7944598; doi:10.1186/s12874-021-01242-9)
Supplement: Supplementary file 1 — Additional file 1: Supplemental Table 1. Training and testing statistics for sex-aggregated XGBoost model. [file 12874_2021_1242_MOESM1_ESM.docx]

**Supplemental Material**

**Supplemental Table 1: Training and testing statistics for sex-aggregated XGBoost model**

|  | Training Dataset | | | Testing Dataset | | |
| --- | --- | --- | --- | --- | --- | --- |
| Fold | Count | RMSE | Bias | Count | RMSE | Bias |
| 1 | 54671 | 4.47 | 0.000 | 6074 | 4.73 | -0.046 |
| 2 | 54671 | 4.48 | -0.001 | 6074 | 4.65 | -0.022 |
| 3 | 54671 | 4.48 | 0.000 | 6074 | 4.62 | -0.038 |
| 4 | 54671 | 4.47 | 0.000 | 6074 | 4.76 | 0.012 |
| 5 | 54671 | 4.48 | 0.000 | 6074 | 4.73 | -0.002 |
| 6 | 54671 | 4.48 | 0.001 | 6074 | 4.66 | 0.086 |
| 7 | 54671 | 4.47 | 0.002 | 6074 | 4.73 | -0.029 |
| 8 | 54671 | 4.47 | 0.001 | 6074 | 4.69 | 0.020 |
| 9 | 54671 | 4.48 | 0.002 | 6074 | 4.67 | 0.052 |
| 10 | 54671 | 4.47 | 0.002 | 6074 | 4.74 | -0.007 |
