# Supplementary material for: Waist circumference prediction for epidemiological research using gradient boosted trees
Source: BMC Med Res Methodol. 2021 Mar 9;21:47. doi: 10.1186/s12874-021-01242-9 (PMC7944598; doi:10.1186/s12874-021-01242-9)
Supplement: Supplementary file 2 — Additional file 2: Supplemental Figure 1. Example of one decision tree (out of 1000) in the first iteration of the gender-aggregated XGBoostmodel. Nodes contain cut-offs for the variable of interest and leaves contain output values. [file 12874_2021_1242_MOESM2_ESM.pdf]

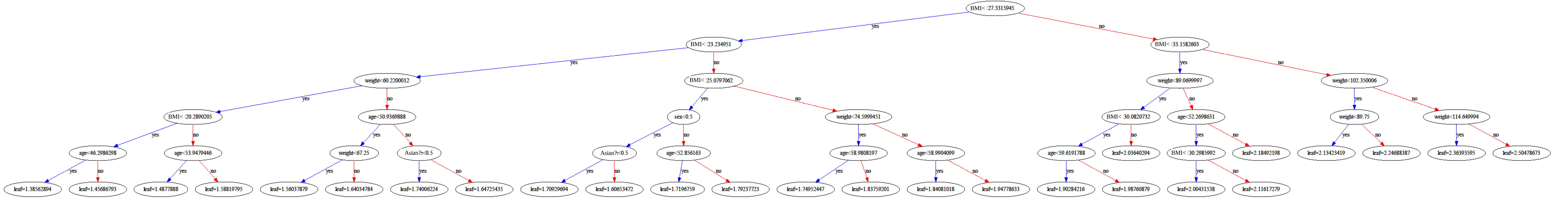

Supplemental Figure 1: Example of one decision tree (out of 1000) in the first iteration of the gender-aggregated XGBoost model. Nodes contain cut-offs for the variable of interest and leaves contain output values.
